# β-Adrenoceptor-mediated Relaxation of Urinary Bladder Muscle in β_2_-Adrenoceptor Knockout Mice

**DOI:** 10.3389/fphar.2016.00118

**Published:** 2016-05-09

**Authors:** Stefan Propping, Kristina Lorenz, Martin C. Michel, Manfred P. Wirth, Ursula Ravens

**Affiliations:** ^1^Department of Urology, Faculty of Medicine Carl Gustav Carus, Dresden University of TechnologyDresden, Germany; ^2^Department of Physiology, Faculty of Medicine Carl Gustav Carus, Dresden University of TechnologyDresden, Germany; ^3^Department of Pharmacology and Toxicology, Julius Maximilian University WürzburgWürzburg, Germany; ^4^Leibniz-Institute für Analytische Wissenschaften-ISAS-e.V.Dortmund, Germany; ^5^West German Heart and Vascular Center Essen, University Hospital Essen-DuisburgDuisburg, Germany; ^6^Department of Pharmacology, Johannes Gutenberg UniversityMainz, Germany

**Keywords:** detrusor muscle, relaxation, mucosa, β_2_-adrenoceptor knockout, β_3_-adrenoceptors, isoprenaline, CL 316,243

## Abstract

**Background and Objective:** In order to characterize the β-adrenoceptor (AR) subtypes involved in agonist-stimulated relaxation of murine urinary bladder we studied the effects of (-)-isoprenaline and CL 316,243 on tonic contraction and spontaneous contractions in detrusor strips of wild-type (WT) and β_2_-AR knockout (β_2_-AR KO) mice.

**Materials and Methods:** Urinary bladders were isolated from male WT and β_2_-AR KO mice. β-AR subtype expression was determined with quantitative real-time PCR. Intact muscle strips pre-contracted with KCl (40 mM) were exposed to cumulatively increasing concentrations of (-)-isoprenaline or β_3_-AR agonist CL 316,243 in the presence and absence of the subtype-selective β-AR blockers CGP 20712A (β_1_-ARs), ICI 118,551 (β_2_-ARs), and L748,337 (β_3_-ARs).

**Results:** Quantitative real-time PCR confirmed lack of β_2_-AR expression in bladder tissue from β_2_-AR KO mice. In isolated detrusor strips, pre-contraction with KCl increased basal tone and enhanced spontaneous activity significantly more in β_2_-AR KO than in WT. (-)-Isoprenaline relaxed tonic tension and attenuated spontaneous activity with similar potency, but the concentrations required were two orders of magnitude higher in β_2_-AR KO than WT. The concentration-response curves (CRCs) for relaxation were not affected by CGP 20712A (300 nM), but were shifted to the right by ICI 118,551 (50 nM) and L748,337 (10 μM). The -logEC_50_ values for (-)-isoprenaline in WT and β_2_-AR KO tissue were 7.98 and 6.00, respectively, suggesting a large receptor reserve of β_2_-AR. (-)-CL 316,243 relaxed detrusor and attenuated spontaneous contractions from WT and β_2_-AR KO mice with a potency corresponding to the drug’s affinity for β_3_-AR. L743,337 shifted the CRCs to the right.

**Conclusion:** Our findings in β_2_-AR KO mice suggest that there is a large receptor reserve for β_2_-AR in WT mice so that this β-AR subtype will mediate relaxation of tone and attenuation of spontaneous activity under physiological conditions. Nevertheless, upon removal of this reserve, β_3_-AR can also mediate murine detrusor relaxation.

## Introduction

Standard therapy of overactive bladder syndrome consists of muscarinic receptor antagonists, but β_3_-AR agonists have recently been introduced as a promising alternative (Chapple et al., 2014). Experimental studies of β-AR-mediated relaxation in isolated detrusor strips are complicated by species differences. While such relaxation of human detrusor is mediated predominantly if not exclusively by the β_3_-AR (for reference, see Wuest et al., 2009), most studies in rats have reported an involvement of both β_2_- and β_3_-AR (Takeda et al., 2003; Uchida et al., 2005). Subtypes involved in mouse bladder are controversial. While we have found that detrusor relaxation is mediated via β_2_-AR (Wuest et al., 2009; Propping et al., 2015a), other authors have suggested β_3_-ARs as the relevant subtype (Deba et al., 2009). Some of this discrepancy may be due to different experimental conditions, but another major issue is that the various drugs employed may actually not exhibit the assumed β-AR subtype selectivity (Cernecka et al., 2014).

Irrespective of the debate on β-AR subtypes involved in detrusor relaxation in various species, it has been questioned whether a direct effect on detrusor smooth muscle cell tone indeed is the underlying cellular mechanism for *in vivo* inhibition of detrusor overactivity by β-AR agonists (Eastham et al., 2015). This is based on the observation that concentrations of β_3_-AR agonists as for instance mirabegron to induce human detrusor strip relaxation are considerably higher (EC_50_ ∼1.7 μM, Svalo et al., 2013) than the plasma concentrations at therapeutic doses (30–75 nM, Krauwinkel et al., 2012). There is some evidence, that modulation of spontaneous contractions could represent an alternative target for the therapeutic effect of β_3_-AR agonists in overactive bladder syndrome. Pre-contracting isolated detrusor tissue with KCl or muscarinic agonists not only increases tonic tension but also induces irregular force oscillations of variable amplitude and frequency (spontaneous contractions, also referred to as “phasic contractions” or “microcontractions”; Gillespie et al., 2015a) Interestingly, spontaneous contractions of detrusor in rats are more sensitive to suppression by (-)-isoprenaline than nerve-mediated contractions evoked by electric field stimulation, but this may be mediated via β_1_-AR (Gillespie et al., 2015b). β-AR subtypes mediating inhibition of spontaneous contractions in other species including mice have not been explored in a systematic manner.

Therefore, we have examined which β-AR subtype mediates inhibition of murine detrusor tone and spontaneous contractions. To address this, we have used the general β-AR agonist (-)-isoprenaline and the β_3_-AR agonist CL 316,243 in KCl-precontracted strips of β_2_-AR knockout (β_2_-AR KO) mice and their wild-type (WT) controls with separate analysis of detrusor tone and spontaneous contractions. Our results confirm the importance of β_2_-ARs for murine detrusor relaxation and attenuation of spontaneous contractions, but also attest contribution of β_3_-ARs.

## Materials and Methods

The control experiments of the present study were performed in FVB/N-WT mice, which match the genetic background of β_2_-AR KO mice. The mice were bred in the Department of Pharmacology and Toxicology, University of Würzburg, Germany. All experiments were performed in accordance with the local authorities (permission number 24D-9168.24-1/2007-17 of the Regierungspräsidium Dresden and of the Regierung of Unterfranken permission number 55.2-2531.01-60/13, Germany) and comply with the European Commission Directive 86/609/EEC regarding the protection and welfare of animals used for experimental as well as scientific purposes.

### Determination of β-AR Subtypes Expression in Mouse Detrusor

Male FVB/N-WT controls and β_2_-AR KO mice (24–40 weeks) were killed by cervical dislocation under CO_2_ anesthesia, and urinary bladders and lungs were removed. The bladders were cut open and detrusor tissue and mucosa were dissected with sharp scissors and further processed separately. RNA was isolated from the tissue samples using the RNeasy^®^-Kit (Qiagen) and total RNA was reverse transcribed using Superscript II reverse transcriptase (Invitrogen). For cDNA amplification of β_1_-ARs, β_2_-ARs, β_3_-ARs and glyceraldehyde-3-phosphate dehydrogenase (GAPDH), a reaction mixture was used containing SsoFast EvaGreen Supermix (BioRad). Quantitative real-time PCR was performed using the C1000 Thermal Cycler CFX96 (BioRad) and the data was analyzed as previously described (Vidal et al., 2012). Amplification conditions for RT-PCR were 15 s at 95°C followed by five cycles of 30 s at 94°C, 30 s at 60°C (for Adrb2 at 64°C) and 30 s at 72°C, five cycles of 30 s at 94°C, 30 s at 62°C and 30 s at 72°C and 25 cycles of 30 s at 94°C, 30 s at 64°C and 30 s at 72°C and an additional cycle of 15 s at 80°C. The following primers were used (Evans et al., 1999; Chernogubova et al., 2005; Wuest et al., 2009; Vidal et al., 2012):

GAPDH forward primer 5′-TGGCAAAGTGGAGATTG TTG-3′;
GAPDH reverse primer 5′-CATTATCGGCCTTGACTG TG-3′;
β_1_ -AR forward primer 5′-CCGCTGCTACCACGACCC CAAG-3′;
β_1_ -AR reverse primer 5′-AGCCAGTTGAAGAAGAG CAAGAGGCG-3′;
β_2_ -AR forward primer 5′-GGTTATCGTCCTGGCCAT CGTGTTTG-3′;
β_2_ -AR reverse primer 5′-TGGTTCGTGAAGAAGTCA CAGCAAGTCTC-3′;
β_3_ -AR forward primer 5′-TCTAGTTCCCAGCGGAGTT TTCATCG-3′;
β_3_ -AR reverse primer 5′-CGCGCACCTTCATAGCCAT CAAACC-3′.

### Experimental Procedure for Measureing Detrusors Contractions and Relaxations

Characterization of mice: the WT and β_2_-AR KO mice had average body weights of 29 ± 3 g for WT (*n* = 55) and 28 ± 4 g for β_2_-AR KO mice (*n* = 49). Strips of mouse urinary bladder were dissected as described previously (Propping et al., 2015a,b). Muscle strips with an intact mucosal layer (mean weight WT mice 2.9 ± 1.6 mg, *n* = 75 strips; β_2_-AR KO mice 2.4 ± 1.8 mg, *n* = 74, *P* = 0.26) were mounted in an organ bath filled with 5 ml of modified Tyrode solution of the following composition (in mM): NaCl 126.9, KCl 5.4, MgCl_2_ 1.05, CaCl_2_ 1.8, NaH_2_HPO_4_ 0.45, NaHCO_3_ 22, EDTA 0.04, ascorbic acid 0.2, glucose 5.6. Phentolamine (3 μM) and prazosin (1 μM) were added to block α-ARs. The solution in the bath was maintained at 37°C, and was oxygenated by vigorously bubbling with carbogen (95% O_2_, 5% CO_2_). The pH was 7.4. All drugs were obtained from the same sources and dissolved either in distilled water of dimethyl sulfoxide as in our previous study (Propping et al., 2015c). The DMSO concentration in the bath did not exceed 0.3%.

The detrusor strips were connected to an isometric force transducer (GM2; Föhr Medical Instruments, Seeheim/Ober-Beerbach Germany) and preloaded with 5 mN. After 30 min in the organ bath, tension was readjusted to 5 mN. Force of contraction was recorded with Chart 4.0TM (AD Instruments, Sydney, NSW, Australia). Tonic tension was analyzed as the increase of force produced by 40 mM KCl, measured from the lower limit of the “noise” produced by spontaneous activity under baseline conditions and in the presence of KCl. The amplitudes and the time integral of spontaneous contractions were analyzed during the 2-min period before the next concentration increase, using Chart software. Agonist-induced attenuation of spontaneous activity was expressed as integral in percent of control.

The preparations were allowed to equilibrate for at least 60 min. During this period, they were stimulated two consecutive times with KCl (40 mM, without osmotic compensation). After another 45 min of washout, the strips were pre-contracted by depolarization with 40 mM KCl. Relaxation was induced with cumulatively increasing concentrations of (-)-isoprenaline, CL 316,243 or forskolin. Relaxation was measured as the difference between minimum force prior to addition of agonist (steady state force) and force in the presence of the agonist, and was expressed in percent of the response to 10 μM forskolin added at the end of each experiment (= 100%). All β-AR subtype-selective blockers were added 30 min before the start of KCl pre-contraction and remained in the bath solution throughout the remainder of the experiment. The concentrations of antagonists were CGP 20712A 300 nM, ICI 118,551 50 nM (Wuest et al., 2009; Propping et al., 2015a), and L748,337 100 nM to 10 μM (Deba et al., 2009).

### Calculation of -logEC_50_ Values

Concentration-response curves were constructed by non-linear regression for each individual experiment by using Prism 5.0^®^(GraphPad^®^Software, Inc., San Diego, CA, USA). The negative logarithm to the base of 10 of the molar concentration producing 50% of the maximum response (-logEC_50_ [M]) as well as the maximum response (E_max_) were calculated and expressed as mean ± SD. Please note that the non-linear regression curves depicted in the figures were fitted to the mean values of the data.

In Schild plots, log(CR-1) was plotted versus log(molar concentration of antagonist), where CR stands for concentration ratio, i.e., the agonist concentration producing 50% of the maximum effect (EC_50_) in the presence of the antagonist divided by the EC_50_ of in the absence of antagonist (Schild, 1947).

The pA_2_ value as a measure of potency of a surmountable antagonist was extrapolated from a straight line with the slope of unity, given by the formula

$$pA_{2}=\log(CR-1)-\log(antagonist\,\;concentration).$$

The same formula was used for calculating the apparent pA_2_ values by substituting the experimental values for one concentration only.

### Statistical Analysis

The results are represented as mean ± standard deviation (mean ± SD). A two-tailed *t-*test for unequal samples with different variances was used for two-group comparisons and was calculated with Prism 5.0^®^(GraphPad^®^Software, Inc., San Diego, CA, USA). Analysis of variance (ANOVA) was used for multiple group comparison, followed by an additional Bonferroni comparison test where appropriate. *P* < 0.05 was regarded as significant.

## Results

### Expression of β-AR Subtypes in Intact Murine Detrusor Muscle

**FIGURE 1:**
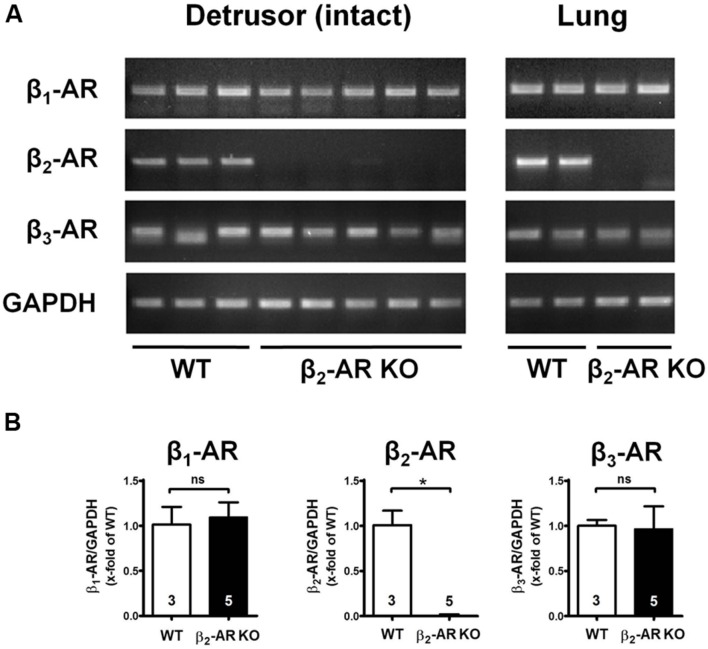
**Expression of β-AR subtypes in urinary bladders und lungs of WT and β_2_-AR knockout mice.** **(A)** Agarose gel electrophoresis of qualitative PCR of cDNA obtained from urinary bladder detrusor samples (left panel) and lung samples (right panel) of WT and β_2_-AR KO mice for β_1_-AR, β_2_-AR, β_3_-AR and glyceraldehyde-3-phosphate dehydrogenase (GAPDH). **(B)** mRNA expression of β_1_-AR, β_2_-AR, and β_3_-AR normalized to GAPDH in detrusor (WT, *n* = 3; β_2_-AR KO, *n* = 5). n represent numbers of mice measured in triplicates. Mean values ± SD.

Expression of the three β-AR subtypes was determined by RT-PCR and quantitative real-time PCR in intact detrusor tissue from WT and genetically modified animals in order to verify knock-out of β_2_-ARs and to check for any compensatory changes in expression of β_1_- and β_3_-ARs. **Figure 1** shows that β_2_-ARs were only detectable in detrusor tissue from WT but not from β_2_-AR KO mice, and the same results were obtained in the respective lung tissues, which served as controls. Furthermore, β_1_- and β_3_-ARs were expressed in bladder and lung tissue from all animals (**Figure 1A**). Between WT and β_2_-AR KO mice there were no differences in expression levels of β_1_- and β_3_-ARs (**Figure 1B**).

### Baseline and KCl-induced Detrusor Contractions in WT and β_2_-AR KO Mice

**FIGURE 2:**
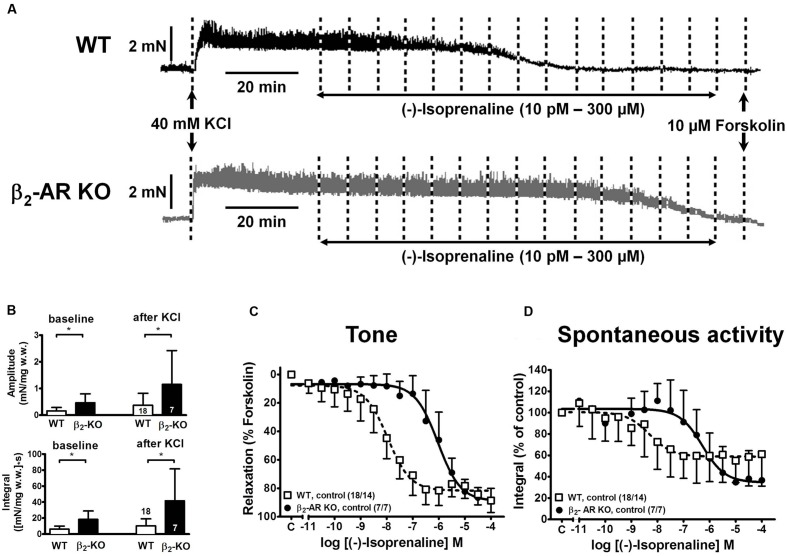
**Relaxation of KCl-precontracted intact detrusor strips from WT and β_2_-AR KO mice.** **(A)** Original tracings of force of contraction (in mN) from two intact detrusor strips precontracted with KCl (40 mM) and subsequently relaxed with cumulatively increasing concentrations of (-)-isoprenaline. Forskolin (10 μM) was added at the end of each experiment. **(B)** Baseline amplitude (upper panel) and baseline integral (lower panel) of spontaneous activity before, and amplitudes and integrals after addition of KCl. **(C)** CRCs for the relaxing effects of (-)-isoprenaline on KCl-induced tone in detrusor strips from WT (open symbols) and β_2_-AR KO (closed symbols). Since tonic contraction was completely relaxed with 10 μM forskolin, the CRCs were normalized to the response with forskolin (= 100%). **(D)** CRCs for spontaneous activity expressed as force-time integral in percent of pre-agonist control for WT (open symbols) and β_2_-AR KO (closed symbols). A quantitative analysis of the data is shown in **Table 1**.

Depolarization of mouse detrusor strips with 40 mM KCl induced a rapid increase in tone and spontaneous activity, which stabilized within 45 min (**Figure 2A**). Mean values of peak force (F_peak_) increases were greater in strips from β_2_-AR KO (2.86 ± 1.34 mN/mg w.w., *n* = 7/7) than WT mice (1.42 ± 0.97 mN/mg w.w., *n* = 18/14; *P* < 0.05); steady-state tone (F_ss_) was 1.57 ± 0.76 mN/mg w.w. β_2_-AR KO and 0.76 ± 0.28 mN/mg w.w. in WT (*P* < 0.05).

In the same detrusor strips the baseline amplitudes of spontaneous contractions and their time integrals were greater in β_2_-AR KO than in WT mice [amplitudes: 0.46 ± 0.34 mN/mg w.w. and 0.16 ± 0.13 mN/mg w.w., *P* < 0.05; integral: 18.34 ± 10.51 (mN/mg w.w.)^∗^s, and 6.26 ± 3.74 (mN/mg w.w.)^∗^s, *P* < 0.05]. After addition of KCl the integral for spontaneous detrusor activity increased significantly to 41.60 ± 39.78 (mN/mg w.w.)^∗^s in β_2_-AR KO and to 10.30 ± 8.77 (mN/mg w.w.)^∗^s in WT (*P* < 0.05; **Figure 2B**).

**FIGURE 3:**
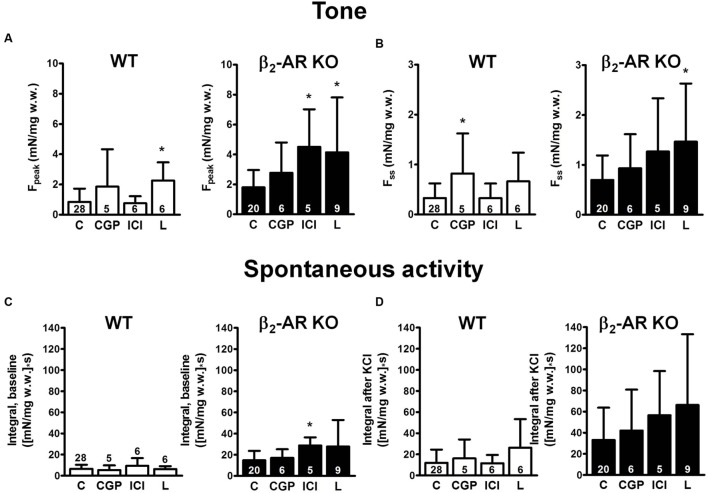
**Effects β-AR blockers on detrusor tone and time-integral of spontaneous activity after exposure to 40 mM KCl (CGP, 300 nM CGP 20712A; ICI, 50 nM ICI 118,551; L748,337, 100 nM L748,337).** **(A,B)** Peak increase (F_peak_) and steady-state increase in tone (F_ss_), respectively, after exposure to 40 mM KCl from WT and β_2_-AR KO preparations. Please note the difference in scale. **(C,D)** Time-integral of spontaneous contractions from WT and β_2_-AR KO preparations; baseline time-integral before **(C)** and time-integral after **(D)** exposure to 40 mM KCl. Mean ± SD from number of experiments as indicated. ^∗^denotes *P* < 0.05 compared with control.

The β_1_-AR blocker CGP 20712A (300 nM) had little effect on KCl-induced F_peak_ and F_ss_ in either mouse strain (**Figure 3**), except for F_ss_ in WT mice (**Figure 3B**). CGP 20712A did not affect spontaneous contraction integral neither at baseline nor after KCl (**Figures 3C,D**). Exposure to the β_2_-AR blocker ICI 118,551 (50 nM) increased F_peak_ and baseline spontaneous activity in strips from β_2_-AR KO mice. Responses to the β_3_-AR blocker L748,337 (100 nM) exhibited great variability, but appeared to increase F_peak_ and F_ss_ rather than spontaneous activity.

### (-)-Isoprenaline-induced Detrusor Relaxation in WT and β_2_-AR KO Mice

Increasing concentrations of (-)-isoprenaline caused almost complete relaxation of tone, and attenuation of spontaneous activity in intact strips from WT and β_2_-AR KO mice (**Figures 2C,D**). However, strips from β_2_-AR KO mice were significantly less sensitive by almost 2 log units. The -logEC_50_ values for (-)-isoprenaline-induced relaxation of tone were 7.98 for WT and 6.00 for β_2_-AR KO mice (see **Table 1**). The respective values for the (-)-isoprenaline-induced attenuation of integral for spontaneous contractions were 8.39 ± 1.06 and 6.34 ± 0.63, and were not significantly different from the effect on relaxation of tone.

**Table 1:** Relaxing effects of (-)-isoprenaline, CL 316,243 and forskolin in murine detrusor strips and their modulation by selective β-AR antagonists.

Mouse strain	Relaxing agent	β-AR antagonist	-logEC_50_ [M]	E_max_ [%]	*n*
WT	(-)-Isoprenaline	None (control)	7.98 ± 0.12	83 ± 2	18/14
		300 nM CGP 20712A	7.89 ± 0.14	88 ± 4	5/4
		50 nM ICI 118,551	6.63 ± 0.15^∗^	96 ± 5^∗^	6/6
		100 nM L748,337	8.06 ± 0.16	88 ± 4	5/5
		1 μM L748,337	7.70 ± 0.50	83 ± 7	4/4
		3 μM L748,337	7.73 ± 0.25	88 ± 2	6/6
		10 μM L748,337	6.49 ± 0.29^∗^	87 ± 3^∗^	5/5
β_2_-AR KO	(-)-Isoprenaline	None (control)	6.00 ± 0.14	94 ± 4	7/7
		300 nM CGP 20712A	5.93 ± 0.11	99 ± 8	6/6
		50 nM ICI 118,551	5.94 ± 0.13	94 ± 5	5/5
		100 nM L748,337	5.74 ± 0.09	94 ± 2	9/9
WT	CL 316,243	None (control)	6.76 ± 0.29	64 ± 12	6/6
		1 μM L748,337	6.02 ± 0.30^∗^	73 ± 9	9/7
		3 μM L748,337	6.40 ± 0.42	90 ± 5^∗^	3/3
		10 μM L748,337	6.17 ± 0.22^∗^	93 ± 2^∗^	4/4
β_2_-AR KO	CL 316,243	None (control)	6.94 ± 0.64	67 ± 16	7/7
		100 nM L748,337	6.44 ± 0.45	66 ± 11	8/8
		300 nM L748,337	6.32 ± 0.74	65 ± 13	7/7
		1 μM L748,337	6.07 ± 0.54	69 ± 10	4/4
		3 μM L748,337	6.25 ± 0.44	87 ± 14	7/7
		10 μM L748,337	6.27 ± 0.58	92 ± 6^∗^	8/8
WT	Forskolin	None (control)	6.63 ± 0.54	–	4/4
β_2_-AR KO	Forskolin	None (control)	6.14 ± 0.24	–	6/6


*Comparison with respective control without any blocker: ^∗^*P* < 0.05. Pre-contraction by 40 mM KCl. -logEC_*50*_, negative logarithm of the concentration of agonist required for half-maximum relaxation in the absence or presence of extra drug; E_*max*_, maximum response to agonist expressed in percent of the response to 10 μM forskolin (= 100%); n, number of detrusor strips in number of mice.*

**FIGURE 4:**
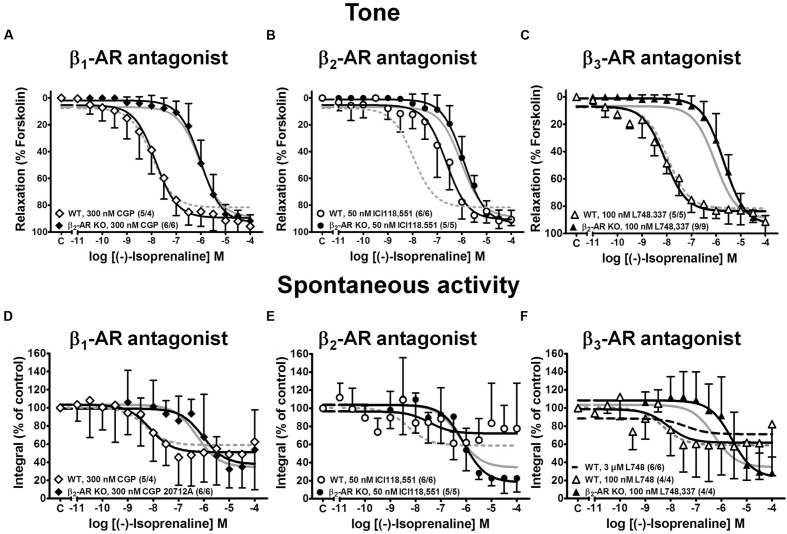
**Effects of various β-AR subtype selective blockers on CRCs for (-)-isoprenaline-induced relaxation of KCl-induced tone **(A–C)** and spontaneous contractions **(D–F)** in detrusor strips from WT (open symbols) and β_2_-AR KO mice (closed symbols).** CRCs were performed in the presence of the β_1_-AR-selective blocker CGP 20712A **(A,D)**, the β_2_-AR-selective blocker ICI 118,551 **(B,E)**, the β_3_-AR-selective blocker L748,337 **(C,F)**. The respective controls (WT, dashed gray lines; β_2_-AR KO, continuous gray lines) were taken from the same experiments as in **Figure 2**. Mean values ± SD from n experiments as indicated in parenthesis (number of strips/number of animals).

In detrusor strips from WT mice, CGP 20712A (300 nM) and L748,337 (100 nM) had little effect on the CRCs of (-)-isoprenaline, whereas the β_2_-AR blocker ICI 118,551 (50 nM) shifted the CRCs to higher (-)-isoprenaline concentrations by about 1.4 log units (**Figure 4**, top panels). In β_2_-AR KO mice, the three β-AR blockers produced little effect on the CRCs of (-)-isoprenaline (**Figures 4A–C**), and the small shift to the right with 100 nM L748,337 did not reach significance. All -logEC_50_ and E_max_ values are summarized in **Table 1**. Spontaneous activity was attenuated by (-)-isoprenaline to a somewhat larger extent in β_2_-AR KO than WT strips (**Figures 4D–F**). The effects of (-)-isoprenaline on spontaneous activity were not influenced by blocking β_1_-AR with CGP 20712A (**Figure 4D**). There was a trend for a shift in the CRC by the β_2_-AR blocker ICI 118,551 in WT strips (**Figure 4E**) and by the β_3_-AR blocker L748,337 in β_2_-AR KO strips (**Figure 4F**). Again, due to the large variability, the effects on spontaneous contractions were less clear than on tonic contraction.

**FIGURE 5:**
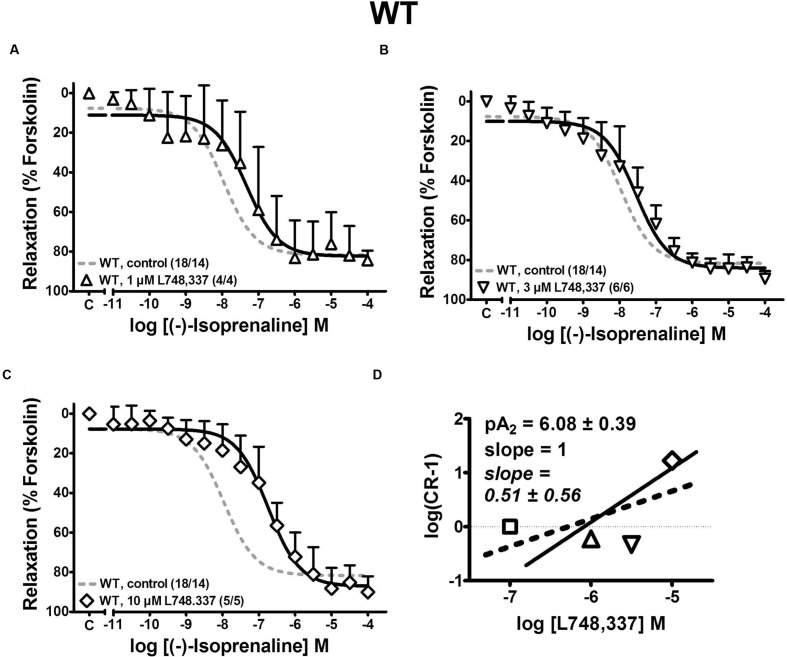
**Effects of the β_3_-AR-selective blocker L748,337, 1 μM **(A)**, 3 μM **(B)**, and 10 μM **(C)**, on CRCs for (-)-isoprenaline-induced relaxation of KCl-precontracted detrusor strips from WT mice.** The control is taken from the same experiments as in **Figure 2**. Mean values ± SD from n experiments as indicated in parenthesis (number of strips/number of animals). **(D)** Schild plot for determining the pA_2_ value of L748,337 (see Materials and Methods for further details).

These findings confirm our previous observations (Wuest et al., 2009; Propping et al., 2013, 2015a) that β_2_-ARs are the major β-AR subtype mediating relaxation in WT detrusor strips. However, given the low affinity of L748,337 for murine β_3_-ARs (Cernecka et al., 2014), the concentration of 100 nM L748,337 may not have been sufficient to block murine β_3_-AR. Therefore, higher L748,337 concentrations were employed to antagonize (-)-isoprenaline-mediated relaxation in WT detrusor (**Figure 5**). With 10 μM L478,337, the CRC was clearly shifted to the right (**Figure 5C**). The results with 1 and 3 μM L748,557 were less consistent (**Figures 5A,B**). Nevertheless, these findings suggest that β_3_-ARs may be involved to a larger extent than previously anticipated by us (Propping et al., 2015a), but as suggested by Deba et al. (2009). The Schild plot (**Figure 5D**) clearly deviated from unity suggesting a more complex mechanism than simple competition of (-)-isoprenaline and L748,337 for a single binding site. Fitting a linear regression of slope 1 to the data points, yielded a pA_2_ value of 6.08 for L748,337 in strips from WT mice.

### CL 316,243-Induced Detrusor Relaxation in WT and β_2_-AR KO Mice

**FIGURE 6:**
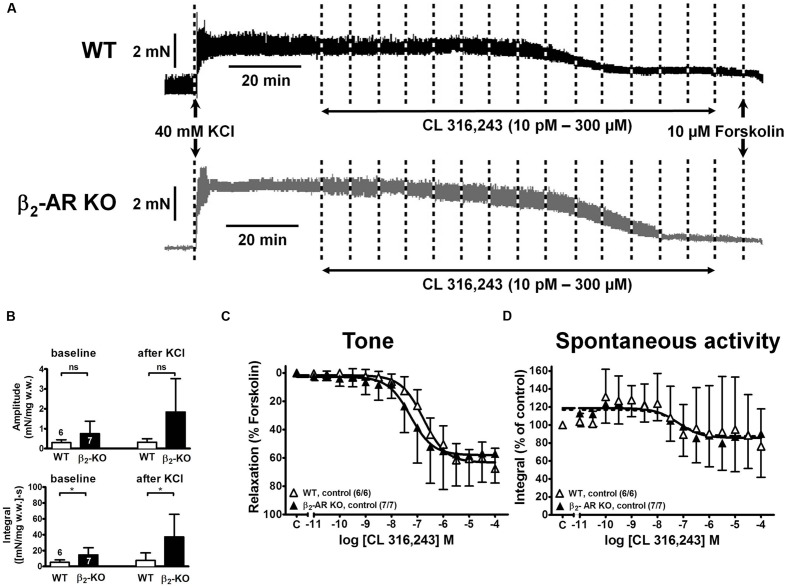
**Relaxation in response to the β_3_-AR-selective agonist CL 316.243 of KCl-pre-contracted detrusor strips from wild type (WT) and β_2_-AR KO mice.** **(A)** Original tracings of force of contraction (in mN) from two detrusor strips precontracted with KCl (40 mM) and subsequently relaxed with cumulatively increasing concentrations of CL 316,243. At the end of each experiment complete relaxation was induced with forskolin (10 μM) as indicated. **(B)** Amplitude (upper panel) and integral (lower panel) of spontaneous activity before and after addition of KCl. **(C,D)** CRCs for the CL 316,243 effects on tone and spontaneous activity in WT and β_2_-AR KO, respectively. Similar lay-out as in **Figure 2**.

**FIGURE 7:**
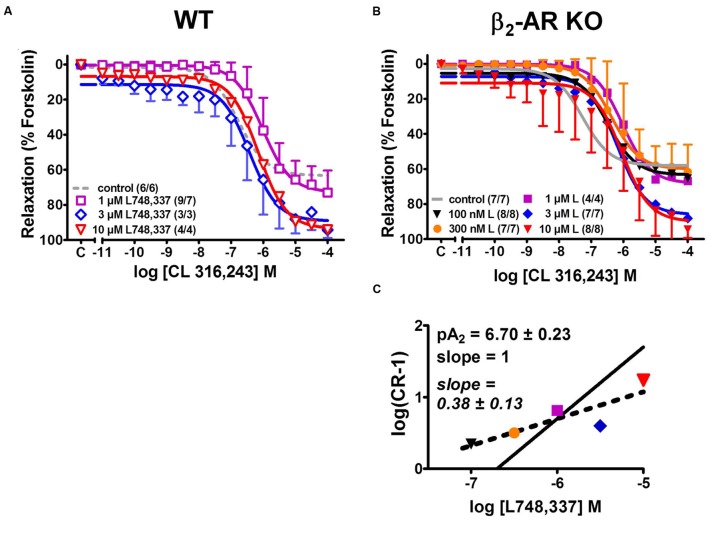
**Effects of various concentrations of the β_3_-AR-selective blocker L748,337 on CRCs for the tone relaxing effects of CL 316,243 in KCl-precontracted detrusor strips from WT **(A)** and β_2_-AR KO mice **(B)**.** Control data (WT, dashed gray lines; β_2_-AR KO, continuous gray lines) were taken from **Figure 2**. **(C)** Schild plot for determining the pA_2_ value of L748,337 in β_2_-AR KO (see Materials and Methods for further details). Please note that no Schild plot could be obtained for data from WT. Mean values ± SD from n experiments as indicated in parenthesis (number of strips/number of animals).

In order to resolve these different interpretations, we next investigated the effects of the β_3_-AR-selective agonist CL 316,243 that was employed by Deba et al. (2009). Increasing concentrations of CL 316,243 relaxed detrusor strips from WT and β_2_-AR KO with similar potency and efficacy (**Figure 6**). In comparison with (-)-isoprenaline, CL 316,243 tended to be less potent in strips from WT than β_2_-AR KO mice (compare **Figure 2**, **Table 1**). Again, more vigorous spontaneous activity was observed in β_2_-AR KO than WT strips (**Figure 6B**). The complete CRCs revealed that detrusor strips relaxed less completely (**Figure 6C**, **Table 1**) and that attenuation of spontaneous activity failed to reach significance both in WT and β_2_-AR KO strips (**Figure 6D**). Concentrations of 100–300 nM L748,337 had no significant effect on the CRCs for CL 316,243 under any experimental condition, but concentrations between 1 and 10 μM L748,337 induced complex changes in relaxation (**Figures 7A,B**). In strips from WT mice, L748,337 did not shift the CRCs of CL 316,243, but with 3 and 10 μM L748,337 relaxation became more complete. In β_2_-AR KO mice, L748,337 caused a shift of the CRCs to higher concentrations of CL 316,243, in addition to more complete relaxation. Again, the Schild plot (**Figure 7C**) deviated from unity indicating a complex mechanism of interaction also between CL 316,243 and L748,337. Using a slope factor of 1, the pA_2_ value was 6.70 for L748,337 in strips from β_2_-AR KO mice.

### β-AR-independent Detrusor Relaxation by Forskolin in WT and β_2_-AR KO Mice

**FIGURE 8:**
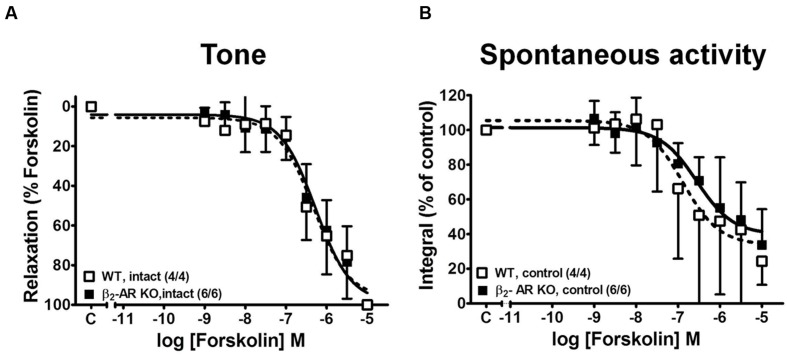
**Concentration-response curves for the relaxing effects of forskolin on KCl-induced contractions in detrusor strips from WT and β_2_-AR KO mice.** **(A)** Forskolin effects on tone; effects on spontaneous contractions **(B)**. Mean values ± SD from n experiments as indicated in parenthesis (number of strips/number of animals).

In order to estimate receptor-independent relaxation we have also studied the responses to adenylyl cyclase activation with forskolin (Morita et al., 1986) in WT and β_2_-AR KO mice (**Figure 8**). Forskolin completely relaxed tonic tension (**Figure 8A**) and attenuated spontaneous contractions (**Figure 8B**) and there were no differences in sensitivity between strips from WT and β_2_-AR KO mice (**Table 1**).

## Discussion

The aim of the present study was to investigate the β-AR subtypes involved in attenuating tonic and spontaneous contraction of murine detrusor in order to elucidate the discrepancy between our own results (Wuest et al., 2009; Propping et al., 2015a) and those of others (Deba et al., 2009) by utilizing β_2_-AR KO mice and β-AR subtype-selective ligands as pharmacological tools (Schmid et al., 2015).

Regulation of detrusor contractility is complex and not fully understood. The central force-developing step in detrusor contraction is the interaction between myosin and actin filaments that occurs upon phosphorylation of myosin light chains (MLC) via Ca^2+^-calmodulin dependent MLC kinase (MLCK, Andersson and Arner, 2004; Hashitani et al., 2004). Several signal transduction pathways are likely to be involved in β-AR-mediated smooth muscle relaxation. The canonical signaling pathway for β-AR involves stimulation of adenylyl cyclase, elevation of cellular cAMP levels and activation of protein kinase A (PKA). Activated PKA phosphorylates MLCK thereby impairing its Ca^2+^-calmodulin-dependent activation, which reduces MLC phosphorylation and hence muscle tone (Andersson and Arner, 2004; Hashitani et al., 2004). β-AR-mediated relaxation also involves Ca^2+^-activated K^+^ channels of large conductance (BK_Ca_ channels; Petkov, 2014). Enhanced BK_Ca_ channel activity via PKA-mediated phosphorylation may contribute to relaxation by hyperpolarizing the cell membrane and reducing Ca^2+^ influx via voltage-dependent Ca^2+^ channels (Wegener et al., 2004). However, Frazier et al. (2005) questioned the role of cAMP in β-AR-mediated relaxation because inhibitors of adenylyl cyclase or PKA had limited effect on rat detrusor relaxation, suggesting that other unknown pathways are involved as well.

### Which β-AR Subtype Mediates (-)-Isoprenaline- or CL 316,243-induced Murine Detrusor Relaxation?

Constitutive systemic knockout of a particular receptor subtype may cause compensatory changes in expression of other receptors or proteins (Michel and Seifert, 2015). Therefore, the expression of all β-AR subtypes in detrusor tissue from WT and transgenic mice was checked using RT- PCR and quantitative real-time PCR. Despite cumulating evidence that GAPDH expression is decreased under conditions of increased sympathetic tone (Michel-Reher and Michel, 2015) we normalized the expression data to GAPDH as a housekeeping gene. In real-time PCR GAPDH remained constant (data not shown) so that we felt safe with this normalization. Our results clearly indicated complete absence of the β_2_-AR subtype, whilst compensatory expression of β_3_-(or β_1_-)ARs is absent in detrusor tissue from β_2_-AR KO mice.

Since the β_2_-AR KO mice were bred against a genetic background different from the previously used C57Bl6 mice, new control experiments had to be performed with FVB/N-WT mice. The results were similar as in C57Bl6 mice (Wuest et al., 2009; Propping et al., 2015a), i.e., the β_2_-AR blocker ICI 118,557 significantly shifted the CRCs for (-)-isoprenaline to the right, confirming that β_2_-ARs are involved in this relaxing effect. The affinity estimate calculated for ICI 118,557 based on these shifts (apparent pA_2_ values: 8.63) was in good agreement with its known affinity at β_2_-AR (for instance 8.92 and 8.8 for human β_2_-AR expressed in CHO cells, respectively, Tate et al., 1991; Palea et al., 2012).

In the absence of any β_2_-ARs in the KO animals, the detrusor relaxation response to (-)-isoprenaline could be mediated by β_1_- or β_3_-AR. Since β_1_-ARs are predominant only in guinea-pig urinary bladder (Yamamoto et al., 1998), the relaxant effect of (-)-isoprenaline in the β_2_-AR KO mice must have been mediated via β_3_-ARs. The (-)-isoprenaline concentrations required for half-maximum relaxation were about ∼90-fold higher in detrusor from β_2_-AR KO than WT mice. Given the affinity of murine β_3_-ARs for (-)-isoprenaline, e.g., -logEC_50_ 5.57 (Blin et al., 1994), this is indeed in the concentration range required for β_3_-AR activation. Furthermore, the selective β_3_-AR agonist CL 316,243 clearly produced relaxation in detrusor strips both from WT and β_2_-AR KO mice with similar -logEC_50_ values which corresponded to the -logEC_50_ value of 8.61 for CL 316,243 at rodent β_3_-AR (Clouse et al., 2007).

The -logEC_50_ value of (-)-isoprenaline for relaxation of WT mouse detrusor, i.e., 7.98 was about 1.5 orders of magnitude lower than its known affinity at mammalian β_2_-AR (pK_i_ 6.4; www.guidetopharmacology.org). This finding suggests that there is a large receptor reserve for detrusor relaxation via β_2_-AR. When β_2_-AR are blocked with ICI 118,551 or are absent as in β_2_-AR KO mice, (-)-isoprenaline is able to relax mouse detrusor via β_3_-AR stimulation, but spare receptors do not appear to play a role in this case.

Although, small molecule inhibitors have been valuable tools to characterize receptor subtypes in pharmacological studies, the interpretation of such results is complicated because the compounds may exhibit non-anticipated receptor-activation patterns or lack of selectivity (Michel and Seifert, 2015). In our previous work, we concluded that the relaxing effect of (-)-isoprenaline in murine detrusor was mediated via β_2_-ARs, because only ICI 118,551 (50 nM) shifted the CRCs for (-)-isoprenaline to the right, whereas the β_1_-AR blocker CGP 20712A (300 nM) and the β_3_-AR blocker L748,337 (100 nM) were without effect (Wuest et al., 2009; Propping et al., 2015a). In contrast, based on experiments with CL 316,243 and with higher concentrations of L748,337 (1–10 μM), other groups reported that murine detrusor relaxes via activation of β_3_-ARs (Deba et al., 2009). Our previous failure to detect a shift in (-)-isoprenaline CRCs by L748,337 (100 nM) in mouse detrusor may in retrospect be explained by the recent observation that L748,337 has 10-100-fold lower affinity to rodent than human β_3_-ARs (Palea et al., 2012; van Wieringen et al., 2013). Some of these species differences in potency have been related to differences in the binding pocket for L748,337 between human and rodent β_3_-AR (Cernecka et al., 2014).

In the present study we employed L748,337 concentrations up to 10 μM and found significant effects on both (-)-isoprenaline- and CL 316,243-induced relaxation of detrusor from WT and β_2_-AR KO mice. In our previous work, Schild plot analysis revealed a surmountable antagonism between ICI 118,551 and (-)-isoprenaline (or adrenaline) in C57Bl6 murine detrusor and between L748,337 and (-)-isoprenaline (or noradrenaline) in human detrusor (Wuest et al., 2009; Propping et al., 2013). However, the mode of antagonism by L748,337 seems to be more complex in the mouse. As expected, the antagonistic effect of L748,337 was most consistent in CL-316,243-stimulated detrusor from β_2_-AR KO mice, i.e., under conditions when relaxation was most likely produced by β_3_-AR activation.

### Effects of β-AR Agonists on Spontaneous Activity

Although, spontaneous activity of detrusor muscle is not fully understood, increasing evidence suggests that smooth muscle cells posses intrinsic mechanisms for spontaneous contractions and that these are synchronized and modulated by interstitial cells distributed throughout the bladder wall (Davidson and McCloskey, 2005; Hashitani, 2006; Lagou et al., 2006). Spontaneous activity in smooth muscle cells and interstitial cells is associated with intracellular Ca^2+^ oscillations but appears to be generated by different mechanism as evidenced by different pharmacological responses (Johnston et al., 2008). Here, we observed larger and more spontaneous c contractions in β_2_-AR KO than WT strips, and in addition, more spontaneous activity developed in β_2_-AR KO strips that were exposed to the β_2_-AR antagonist ICI 118,551. While the former finding could suggest adaptive responses to the chronic absence of β_2_-AR mediated signaling pathways, we do not have a plausible explanation for the latter puzzling finding, which needs to be verified in future studies in order to exclude random variation as an underlying cause. Attenuation of spontaneous contractions by (-)-isoprenaline occurred in the same concentration ranges in WT and β_2_-AR KO strips as relaxation of tonic tension, and the CRC was only shifted to the right by ICI118,551 suggesting a dominant role for β_2_-AR in this process. Nevertheless, the small attenuation of spontaneous activity by CL 316,243 indicates a modulating effect of β_3_-AR as well. Our findings do not confirm that suppression of phasic contractions by (-)-isoprenaline is most sensitive to β_1_-AR blockers (Gillespie et al., 2015b), because we did not observe any shift in CRC with CGP 20712A. Taken together, comparison of the effects of subtype-selective β-AR agonists and antagonists suggests that tonic and spontaneous detrusor contractions may be modulated by different pathways but both β_2_- and β_3_-AR appear to be involved.

### Effects of Forskolin

Relaxation of tonic and phasic detrusor contractions after receptor-independent activation of adenylyl cyclase with forskolin in WT and β_2_-AR KO mice were similar between the two groups. Furthermore, also after forskolin, spontaneous activity was suppressed less completely than tonic tension.

## Conclusion

We have reported an example how false extrapolation of drug affinities for a given receptor subtype from different species can lead to an incomplete picture. Our novel findings in β_2_-AR KO mice suggest that there is a large receptor reserve for β_2_-AR so that this β-AR subtype will be activated preferentially by physiological ligands. Nevertheless β_3_-AR can also mediate relaxation and attenuate spontaneous contractions in the absence of β_2_-AR, when β_2_-AR are blocked or when selective β_3_-AR agonists are used.

## Author Contributions

SP: experimental procedure, result analysis, evaluation of results, writing the manuscript; KL: experimental procedure, result analysis, evaluation of results, writing the manuscript; MM: result analysis, evaluation of results, revision of the manuscript; MW: result analysis, evaluation of results, revision of the manuscript; UR: result analysis, evaluation of results, writing and revision of the manuscript.

## Conflict of Interest Statement

The authors declare that the research was conducted in the absence of any commercial or financial relationships that could be construed as a potential conflict of interest.

## Abbreviations

β-AR: β-adrenoceptor
β_2_-AR KO: β-2-adrenoceptor knockout
CGP 20712A: 1-[2-((3-carbamoyl-4-hydroxy)phenoxy)ethylamino]-3-[4-(1-methyl-4-trifluoromethyl-2-imidazolyl)phenoxy]-2-propanol
CL 316,243: (R,R)-5-[2-[[2-(3-chlorophenyl)-2-hydroxy-ethyl]-amino]propyl]-1,3-benzodioxole-2,2-dicarboxylate
CRC: concentration response curve
ICI 118,551: (±)-1-[2,3-(dihydro-7-methyl-1H-inden-4-yl)oxy]-3-[(1-methylethyl)amino]-2-butanol
(-)-isoprenaline: 4-[1-hydroxy-2-[(1-methylethyl)amino] ethyl]-1,2-benzenediol hydro-chloride
L748,337: (S)-N-[4-[2-[3-[3-(acetamidomethyl) phenoxy]-2-hydroxypropyl]-amino]-ethyl]-phenylbenzsulfonamide)
RT-PCR: reverse transcription polymerase chain reaction
